# Hypertriglyceridemia Is a Potential Preoperative Predictor for Biochemical Recurrence after Radical Prostatectomy

**DOI:** 10.1371/journal.pone.0122438

**Published:** 2015-03-24

**Authors:** Minyong Kang, Chang Wook Jeong, Ja Hyeon Ku, Choel Kwak, Hyeon Hoe Kim

**Affiliations:** Department of Urology, Seoul National University Hospital, Seoul, Republic of Korea; University of Colorado Denver, UNITED STATES

## Abstract

**Objectives:**

Many previous studies have suggested that the outcome of prostate cancer (PCa) may be closely related to abnormal lipid metabolism. Therefore, in this study, we evaluated the preoperative lipid profiles of patients with clinically localized prostate cancer (PCa) who underwent radical prostatectomy (RP), with particular emphasis on the relationship between these profiles and biochemical recurrence (BCR).

**Patients and Methods:**

We evaluated 715 consecutive men with clinically localized PCa who underwent RP at our institution between January 2011 and December 2013. We defined hypertriglyceridemia as a fasting serum triglyceride (TG) level greater than 200 mg/dL. We used the Kaplan—Meier method to predict BCR-free survival and applied the log-rank test to determine the statistical significance between survival curves. Cox proportional hazard ratio (HR) models were used to identify the significant predictors of BCR according to clinicopathological variables.

**Results:**

Of 663 patients who underwent RP for clinically localized PCa, 66 (10.0%) showed BCR during a median follow-up period of 21 months. Patients without BCR had higher levels of serum TG, and patients with hypertriglyceridemia were significantly more likely to achieve BCR-free survival in the Kaplan—Meier analysis (log-rank test, P = 0.009). In the multivariable analysis, the presence of hypertriglyceridemia (HR 0.22), pathologic Gleason score (≥8; HR 2.85), pathologic T stage (≥pT3; HR 3.44), and a positive surgical margin (HR, 2.39) were still significant BCR predictors.

**Conclusions:**

We found that preoperative hypertriglyceridemia was associated with a lower risk of BCR after RP in patients with clinically localized PCa. Our results could help to clarify the currently conflicting evidence on the relationship between serum lipid profiles, particularly the presence of hypertriglyceridemia, and the risk of BCR in PC a patients after surgery.

## Introduction

Cholesterol is a building block and functional unit of the cell membrane, and hence, activated lipogenesis and the intracellular accumulation of cholesterol is a significant metabolic feature of highly proliferating cancer cells [1]. Therefore, the association between a high-fat diet and the risk of cancer development has been of considerable interest and subject to extensive research over the last decade [2,3]. As with other solid tumors, cholesterol homeostasis is disrupted in pre-malignant and cancerous prostate lesions. However, although many epidemiologic studies have suggested that cholesterol plays important roles in the development and progression of prostate cancer (PCa) [4–7], the association between cholesterol levels and PCa is still not very clear.

In this context, serum lipid profiles can be promising biomarkers for PCa like the well-established association between abnormal serum lipid profiles and the risk of cardiovascular diseases [8]. However, the precise relationship between lipid metabolism markers and PCa remains unclear. For example, increased cholesterol levels were found to be associated with a higher risk of aggressive and recurrent PCa in some reports [5,9,10], whereas other studies failed to find a significant relationship between serum cholesterol levels and total PCa risk or mortality [11,12]. In addition, many studies focused on total cholesterol (TC) and its well-established sub fractions, low-density lipoprotein (LDL) cholesterol and high-density lipoprotein (HDL) cholesterol.

In this study we investigated a range of preoperative blood lipid profiles, TC, HDL, triglyceride (TG), apolipoprotein A1 (apoA1), and apolipoprotein B (apoB), as well as the specific ratio of these markers (TC/HDL, LDL/HDL and apoB/apoA1) of patients with clinically localized PCa who underwent radical prostatectomy (RP), particularly with respect to the relationship between these markers and biochemical recurrence (BCR) after surgery.

## Patients and Methods

### Ethics statement

The Institutional Review Board (IRB) of Seoul National University Hospital reviewed and approved the present study (IRB approval number: H-1411–006–620). Because this was a retrospective study, the IRB waived the requirement for written informed consent from patients. Thus, patient information was anonymized and de-identified prior to analysis in the present study.

### Patient population

Our sample included 715 consecutive men with clinically localized PCa who underwent RP at Seoul National University Hospital between January 2011 and December 2013. We excluded patients who were receiving cholesterol-lowering drugs such as statins, neo-adjuvant hormone therapy, showed no reduction of serum prostate-specific antigen (PSA) level to the nadir after RP, underwent immediate adjuvant therapy post-operatively (including anti-androgen treatment and radiotherapy), and/or for whom there were no records of the relevant lipid profiles. After excluding these patients, we finally included 663 patients in the analysis. All patients were followed to examine whether PSA resurged after surgery. According to the standard protocol of our institution, we checked serum PSA level every 3 months. BCR was defined as either two consecutive increases in serum PSA (>0.2 ng/mL) or the administration of adjuvant therapy during the postoperative follow-up period.

### Examination of clinicopathologic characteristics

We reviewed the following clinicopathological parameters: age, serum PSA levels, total prostate volume, body mass index, the presence of hypertension and diabetes mellitus, biopsy, and the final pathologic Gleason score (GS), extra-prostatic extension (EPE) status, seminal vesicle invasion (SVI) status, surgical margin (SM) status, lymph node metastasis (LNM), and the development of BCR. Pathological stages were determined according to the 7th edition of the American Joint Committee on Cancer staging system. According to a standardized processing protocol, we fixed all prostate tissue after surgery in a solution of 10% neutral buffered formalin, and embedded samples in hot wax to form a paraffin block. A thin section (4-mm thick) of the samples was cut from the paraffin block using a microtome, and stained with hematoxylin and eosin for histopathological evaluation. Experienced urologic pathologists then examined the slides microscopically according to a standardized reporting system used at our institution. We measured the serum lipid profiles (TC, HDL, TG, apoA1, and apoB) 1 month or less before radical prostatectomy, by using blood samples obtained from patients who had fasted for at least 8 hours. Briefly, an enzymatic calorimetric test was used to measure TC and TG levels, a selective inhibition method was adopted to examine HDL cholesterol levels, and rate nephelometry was used to determine apoA1 and apoB levels. LDL cholesterol levels were not generally measured in a laboratory setting. Instead, they were estimated from the apoB value, because apoB is the primary structural component of atherogenic cholesterol particles such as LDL [13]. We stratified the patients into two groups according to the presence of hypertriglyceridemia (defined as a fasting serum TG level greater than 200 mg/dL), based on the National Cholesterol Education Program-Adult Treatment Panel III (NCEP-ATP III) criteria [14]. We offered all patients’ clinicopathological information used in the present study as supplementary data (S1 Dataset).

### Statistical analysis

When comparing two different groups, we used the χ^2^ test for categorical variables and the Mann-Whitney *U* test for continuous variables. We used the Kaplan—Meier method to predict BCR-free survival and applied the log-rank test to determine the statistical significance between survival curves. We applied the Cox proportional hazard ratio (HR) model to identify significant predictors of BCR amongst the clinicopathological variables. Two-sided null hypotheses of no difference in BCR-free survival for each variable were rejected if *p*-values were less than 0.05, or, equivalently, if the 95% confidence interval (CI) of the risk point estimate excluded 1. All statistical analyses were performed using the IBM SPSS Statistics 19.0 (SPSS, Inc., an IBM Company, Chicago, Illinois, USA) and GraphPad Prism (GraphPad Software Inc., San Diego, CA, USA).

## Results

The relevant patient characteristics are summarized in Table 1. The median patient age was 68 years [interquartile range (IQR), 12–28 years], and the median serum PSA level was 6.64 ng/mL (IQR, 4.65–11.28), and the median body mass index (BMI) was 24.25 m^2^/kg (IQR, 22.46–26.03 m^2^/kg). Of the 663 patients, 318 (48.0%) had hypertension and 106 (16.0%) had diabetes mellitus. The median values of serum lipid profiles were not included in the NCEP-ATP III criteria of hyperlipidemia [14]. In the pathologic findings, more than 90% of patients had a tumor with a GS ≤ 7. EPE was identified in 221 patients (33.3%), and SVI was detected in 60 patients (9.0%). Only 21 patients (3.2%) were diagnosed with LNM. Sixty-six patients (10%) showed BCR during a median follow-up period of 24.0 months (IQR, 14.0–30.0 months), and the median BCR duration was 23.0 months (IQR, 13.0–30.0 months).

**Table 1:** Patient demographics.

**Clinical parameters**	
Age, years (median [IQR])	68 (62–72)
Body mass index, m^2^/kg (median [IQR])	24.2 (22.4–26.0)
Hypertension, N (%)	318 (48.0)
Diabetes mellitus, N (%)	106 (16.0)
Serum PSA, ng/mL (median [IQR])	6.64 (4.98–11.28)
Total prostate volume, mL (median [IQR])	39.0 (31.6–49.4)
**Serum lipid profiles**	
Total cholesterol, mg/dL (median [IQR])	180.0 (155.0–203.0)
Triglyceride, mg/dL (median [IQR])	124.0 (92.0–173.0)
ApoB, mg/dL (median [IQR])	89.0 (75.0–103.0)
ApoA1, mg/dL (median [IQR])	115.0 (105.0–128.0)
HDL cholesterol, mg/dL (median [IQR])	47.0 (40.0–56.0)
Total cholesterol/HDL (median [IQR])	1.40 (1.01–1.91)
TG/HDL (median [IQR])	2.60 (1.66–4.05)
ApoB1/ApoA1 (median [IQR])	0.77 (0.65–0.90)
**Pathological parameters**	
Gleason score at biopsy, N (%)	
≤6	275 (45.0%)
7	238 (38.9%)
8	75 (12.3%)
≥9	23 (3.7%)
Gleason score of the resected specimens, N (%)	
≤6	184 (28.1%)
7	423 (64.6%)
8	19 (2.9%)
≥9	29 (4.4%)
Extracapsular extension, N (%)	221 (33.3%)
Seminal vesicle invasion, N (%)	60 (9.0%)
Positive surgical margin, N (%)	193 (29.1%)
Lymph node metastases, N (%)	21 (3.2%)
**Follow-up parameters**	
Biochemical recurrence, N (%)	66 (10.0%)
BCR duration (median [IQR])	23.0 (13.0–30.0)
Follow-up duration, months (median [IQR])	24.0 (14.0–30.0)

Abbreviations: IQR, interquartile ratio; PSA, prostate-specific antigen; ApoB, apolipoprotein B; ApoA1, apolipoprotein A1; HDL, high-density lipoprotein; TG, triglyceride; BCR, biochemical recurrence.

**Fig 1:**
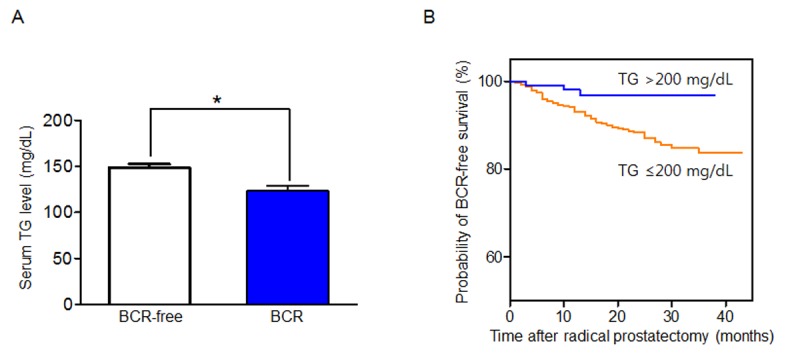
The association between serum triglyceride (TG) levels and biochemical recurrence (BCR). (A) Comparison of the serum TG levels after radical prostatectomy with respect to BCR. (B) The Kaplan—Meier analysis for BCR-free survival according to the presence of hypertriglyceridemia (>200 mg/dL). The log-rank test was performed to determine the statistical significance between the two groups.

We investigated whether the BCR rate was different with respect to the preoperative lipid profiles. Among various parameters, only serum TG level was associated with the BCR rate. When we categorized serum TG levels into non-hypertriglyceridemia (≤200 mg/dL) and hypertriglyceridemia (>200 mg/dL) groups, patients with hypertriglyceridemia had significantly lower BCR rate compared to those without hypertriglyceridemia (Table 2). As shown in Fig. 1A, patients with BCR also had significantly lower serum TG levels compared to those without BCR after surgery. Additionally, patients with hypertriglyceridemia had slightly younger age and higher BMI, as well as the higher levels of TC and apoB, and the lower levels of HDL cholesterol (Table 2). However, all pathologic characteristics did not differ between patients according to whether they had preoperative hypertriglyceridemia despite different BCR rates. Furthermore, the Kaplan—Meier analysis showed that the patients with hypertriglyceridemia were significantly more likely to achieve BCR-free survival than those without hypertriglyceridemia (Fig. 1B; log-rank, *P* = 0.009), with a 3-year BCR-free survival rate of 97.8% (95% CI, 96.2–99.4) and 86.7% (95% CI, 84.6–88.8), respectively.

**Table 2:** Patient characteristics according to serum TG levels.

	Non-hypertriglyceridemia (N = 553)	Hypertriglyceridemia (N = 109)	*P* value
**Clinical parameters**			
Age, year (median [IQR])	68 (62–72)	66 (60–70)	0.009
Body mass index, m^2^/kg (median [IQR])	24.0 (22.3–25.9)	25.3 (23.5–26.7)	<0.001
Hypertension, N (%)	266 (48.0)	52 (47.7)	0.519
Diabetic mellitus, N (%)	83 (15.0)	23 (21.1)	0.076
Serum PSA, ng/mL (median [IQR])	6.61 (4.98–11.40)	6.64 (4.88–10.14)	0.544
Total prostate volume, mL (median [IQR])	39.0 (31.8–49.7)	38.1 (30.7–48.8)	0.666
**Serum lipid profiles**			
Total cholesterol, mg/dL (median [IQR])	173.0 (151.0–198.0)	190.0 (169.0–211.0)	<0.001
Triglyceride, mg/dL (median [IQR])	111.0 (86.0–148.0)	255.0 (220.0–325.0)	<0.001
ApoB, mg/dL (median [IQR])	88.0 (74.0–102.0)	94.0 (82.0–109.0)	0.005
ApoA1, mg/dL (median [IQR])	115.0 (105.5–128.0)	112.0 (103.0–124.0)	0.089
HDL cholesterol, mg/dL (median [IQR])	49.0 (42.5–58.0)	39.0 (35.0–45.0)	<0.001
Total cholesterol/HDL (median [IQR])	1.57 (1.18–2.03)	0.71 (0.53–0.86)	<0.001
TG/HDL (median [IQR])	2.23 (1.54–3.21)	6.50 (5.08–9.16)	<0.001
ApoB/ApoA1 (median [IQR])	0.75 (0.64–0.89)	0.84 (0.71–0.99)	<0.001
**Pathological parameters**			
Gleason score at biopsy, N (%)			
≤ 7	425 (83.5)	88 (86.3)	0.297
≥ 8	84 (16.5)	14 (13.7)	
Gleason score at specimens, N (%)			
≤ 7	506 (92.7)	103 (94.5)	0.329
≥ 8	40 (7.3)	6 (5.5)	
Pathologic T stage			
≤ pT2	374 (67.6)	66 (61.1)	0.115
≥ pT3	179 (32.4)	42(38.9)	
Lymph node metastases, N (%)	19 (5.2)	2 (2.6)	0.256
Positive surgical margin, N (%)	164 (29.7)	29 (27.1)	0.338
Biochemical recurrence, N (%)	63 (11.4)	3 (2.8)	0.002

Abbreviations: TG, triglyceride; IQR, interquartile ratio; PSA, prostate-specific antigen; ApoB, apolipoprotein B; ApoA1, apolipoprotein A1; HDL, high-density lipoprotein; TG, triglyceride.

We next examined the various preoperative variables to determine if any were predictive factors for BCR after RP, focusing in particular on the preoperative status of serum lipid profiles (Table 3). In the univariate analysis, several parameters were identified as BCR predictors, including serum PSA, TG (both continuous and categorical), biopsy GS (≤ 7 vs ≥ 8), pathologic GS (≤ 7 vs ≥ 8), pathologic T stage (≤ pT2 vs ≥ pT3), the presence of LNM, and the presence of positive SM. Of these factors, the presence of hypertriglyceridemia (>200 mg/dL) (HR 0.22, 95% CI 0.05–0.91), advanced pathologic GS (≥8) (HR 2.85, 95% CI 1.42–5.74), advanced pathologic T stage (≥pT3) (HR 3.44, 95% CI 1.65–7.12), and a positive SM (HR 2.39, 95% CI 1.26–4.56) were identified as BCR predictors in the multivariate analysis.

**Table 3:** Cox proportional hazard ratio model to identify predictive factors for BCR after radical prostatectomy.

Preoperative variables	Univariate			Multivariate		
	HR	95% CI	*P* value	HR	95% CI	*P* value
Serum PSA level (continuous)	1.01	1.00–1.02	0.024	-	-	-
Serum TG level (continuous)	0.99	0.99–1.00	0.032	-	-	-
Serum TG level (categorical)						
≤200 mg/dL (non-hypertriglyceridemia)	Reference			Reference		
>200 mg/dL (hypertriglyceridemia)	0.24	0.07–0.77	0.016	0.22	0.05–0.91	0.037
Biopsy GS						
≤7	Reference			Reference		
≥8	2.43	1.39–4.24	0.002	-	-	-
Pathologic GS						
≤7	Reference			Reference		
≥8	4.42	2.52–7.76	<0.001	2.85	1.42–5.74	0.003
Pathologic T stage						
≤pT2	Reference			Reference		
≥pT3	3.65	2.22–6.01	<0.001	3.44	1.65–7.12	0.001
LNM						
Negative	Reference			Reference		
Positive	4.81	2.25–10.27	<0.001	-	-	-
Surgical margin						
Negative	Reference			Reference		
Positive	3.69	2.26–6.04	<0.001	2.39	1.26–4.56	0.008

Abbreviations: BCR, biochemical recurrence; PSA, prostate-specific antigen; TG, triglyceride; GS, Gleason score; LNM, lymph node metastasis; HR, hazard ratio; CI, confidence interval

## Discussion

There is growing evidence that cholesterol plays a role in PCa, and a number of recent studies have suggested that it functions in PCa development and progression. Zhuang *et al*.[15] provided evidence that cholesterol-mediated signaling pathways are associated with cancer cell survival and tumor progression. Llaverias *et al*.[16] showed that hypercholesterolemia, induced by a Western-type diet, accelerated tumor growth and progression in a mouse model of PCa, and a PCa-related function was further supported by epidemiological findings [17–19]. Platz and colleagues noted that PCa patients with low cholesterol were less likely to have a high-grade tumor [4], and a large prospective study by Kitahara *et al*. revealed that a higher TC level was positively associated with the risk of PCa development compared to a lower TC level [20].

Among several cholesterol carrier complexes, TG is a major component of very-low-density lipoprotein and chylomicron, and is both a crucial energy source and a delivery system for dietary fats. High serum TG levels are associated with an increased risk of obesity, atherosclerosis, and cardiovascular diseases. Additionally, many studies have found a significant association between high serum TG levels and the risk of cancer development. However, despite the well-known association of fat intake and the risk of PCa, the impact of TG accumulation in PCa continues to be debated. Hayashi *et al*.[21] showed that high serum TG levels (≥150 mg/dL) significantly increased the risk of both developing PCa and of having a tumor with aggressive histology (GS ≥ 8), particularly in patients aged over 60 years. A recent study by Allot *et al*. showed that elevated serum TG levels (≥150 mg/dL) were significantly associated with BCR in PCa patients who underwent RP (HR 1.35). These findings may be explained by the potential mechanisms of hypertriglyceridemia-induced chronic stress, which include insulin resistance, inflammation, and oxidative stress [22].

In contrast to previous reports, patients without hypertriglyceridemia had a higher risk of BCR, while pathologic characteristics did not differ between patients according to whether they had hypertriglyceridemia in our study. Moreover, it was particularly noteworthy that preoperative hypertriglyceridemia was significantly associated with a reduced risk of BCR (reduced by 80% [HR, 0.22]) after RP, based on the multivariate analysis. This was further supported by the Kaplan—Meier analysis, which revealed a reduced risk of BCR in patients with hypertriglyceridemia. A number of metabolic pathways are involved in cancer development, most notable of which is a shift from a catabolic to an anabolic state [23]. Cancer cells can also reduce serum cholesterol levels, which could provide a mechanistic basis for our findings [24]. Malignant cells grow and proliferate rapidly, and exhibit accelerated metabolic turnover, requiring large amounts of cholesterol for the biosynthesis of new cell membrane [25]. This in turn could result in lower serum cholesterol levels. Additionally, certain type of cancer such as renal cell carcinoma (RCC) has elevated amounts of intracellular lipids for accelerated biosynthesis of cell membrane during rapid proliferation. Recent study by Martino *et al*.[26] reported that preoperative total cholesterol can be an independent predictive factor for survival in RCC patients treated with partial or radical nephrectomy. Particularly, patients with high levels of preoperative cholesterol had better oncologic outcomes. Ko and colleagues also noted that patients with higher preoperative cholesterol levels showed better progression-free survival compared to those with lower cholesterol levels [27]. In this regard, we believe that patients with poor prognosis may have lower baseline cholesterol due to abundant storage of lipids in their cancer cells. In order to provide more convincing evidence to our conclusion, further studies are required to validate whether intracellular cholesterol and TG significantly accumulated in the surgical specimens of PCa patients showing BCR.

Our study has several limitations that need to be considered. The first is its retrospective nature. Second, there is a critical concern with respect to the single measurement of lipid profiles throughout the study period. To determine whether the lipid levels of patients remain the same as that obtained preoperatively, repeated measurements of various lipid markers would be required in a future study. Third, we only started testing specific parameters in lipid panels, such as apolipoproteins, in our institution since 2011. We therefore could only examine the patients’ data obtained between 2011 and 2013. Thus, the study had only a short follow-up duration, resulting in a lower BCR rate compared to previous studies, and preventing us from assessing oncologic outcomes such as progression-free survival and overall survival. Fourth, we did not evaluate serum TG levels after RP and cannot determine whether postoperative serum TG levels can also predictive for BCR. Finally, we did not examine the patients’ overall nutrient status, the pattern of dietary intake, and the type of medications used for controlling diabetes and hypertension, which could also affect the prognosis of PCa. Nevertheless, the present study helps to resolve the controversial question of whether there is an association between serum lipid profiles, particularly TG levels, and the risk of BCR after surgery.

## Conclusions

In summary, patients with hypertriglyceridemia had a better BCR-free survival rate than those without hypertriglyceridemia, and the presence of hypertriglyceridemia, advanced pathologic GS (≥8), advanced pathologic T stage (≥pT3) and a positive SM were predictive factors for BCR in the multivariate analysis. Therefore, our results suggest that preoperative serum TG status, particularly the presence of hypertriglyceridemia, can be a potential predictor for biochemical failure after RP in patients with clinically localized PCa.

## Supporting Information

S1 Dataset All clinicopathological information used in this study. (XLSX)
